# Di-μ-chlorido-bis­({8-[bis­(naphthalen-1-yl)phosphan­yl]naphthalen-1-yl-κ^2^ *C* ^1^,*P*}palladium(II)) dichloro­methane disolvate

**DOI:** 10.1107/S1600536812048222

**Published:** 2012-11-30

**Authors:** Wade L. Davis, Alfred Muller

**Affiliations:** a Research Centre for Synthesis and Catalysis, Department of Chemistry, University of Johannesburg (APK Campus), PO Box 524, Auckland Park, Johannesburg, 2006, South Africa

## Abstract

The title compound, [Pd_2_{P(C_10_H_7_)_2_(C_10_H_6_)}_2_Cl_2_]·2CH_2_Cl_2_, shows cyclo­metalation of one naphthalen-1-yl substituent of each of the phosphane ligands to the Pd dimer in a *trans* orientation; the complete dimer is generated by a centre of inversion. Two dichloro­methane solvent mol­ecules create C—H⋯Cl inter­actions with the metal complex, generating supermolecular layers in the *ab* plane. Additional C—H⋯π and π–π [centroid–centroid distances = 3.713 (3), 3.850 (4) and 3.926 (3) Å] inter­actions join these planes into a three-dimensional supermolecular network.

## Related literature

For background to palladium compounds in catalysis, see: Dunina *et al.* (2008 ▶, 2009 ▶); Bedford *et al.* (2004 ▶); Morales-Morales *et al.* (2002 ▶). For the synthesis of the starting materials, see: Drew & Doyle (1990 ▶).
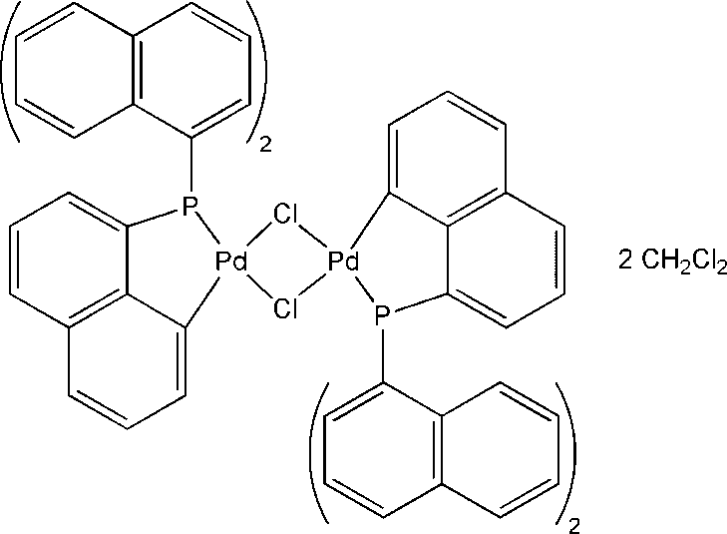


## Experimental

### 

#### Crystal data

[Pd_2_(C_30_H_20_P)_2_Cl_2_]·2CH_2_Cl_2_
*M* *_r_* = 1276.41
Triclinic, 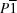

*a* = 9.4823 (8) Å
*b* = 11.4272 (9) Å
*c* = 12.343 (1) Å
α = 80.652 (2)°
β = 76.592 (2)°
γ = 89.013 (2)°
*V* = 1283.42 (18) Å^3^
*Z* = 1
Mo *K*α radiation
μ = 1.12 mm^−1^
*T* = 100 K
0.33 × 0.13 × 0.13 mm

#### Data collection

Bruker APEX DUO 4K-CCD diffractometer
Absorption correction: multi-scan (*SADABS*; Bruker, 2008 ▶) *T*_min_ = 0.709, *T*_max_ = 0.868
40200 measured reflections
6340 independent reflections
5675 reflections with *I* > 2σ(*I*)
*R*_int_ = 0.030

#### Refinement

*R*[*F* ^2^ > 2σ(*F* ^2^)] = 0.052
*wR*(*F* ^2^) = 0.154
*S* = 1.07
6340 reflections
325 parameters
H-atom parameters constrained
Δρ_max_ = 1.59 e Å^−3^
Δρ_min_ = −2.36 e Å^−3^

### 

Data collection: *APEX2* (Bruker, 2011 ▶); cell refinement: *SAINT* (Bruker, 2008 ▶); data reduction: *SAINT* and *XPREP* (Bruker, 2008 ▶); program(s) used to solve structure: *SIR97* (Altomare *et al.*, 1999 ▶); program(s) used to refine structure: *SHELXL97* (Sheldrick, 2008 ▶); molecular graphics: *DIAMOND* (Brandenburg & Putz, 2005 ▶); software used to prepare material for publication: *publCIF* (Westrip, 2010 ▶) and *WinGX* (Farrugia, 2012 ▶).

## Supplementary Material

Crystal structure: contains datablock(s) global, I. DOI: 10.1107/S1600536812048222/zq2190sup1.cif

Structure factors: contains datablock(s) I. DOI: 10.1107/S1600536812048222/zq2190Isup2.hkl

Additional supplementary materials:  crystallographic information; 3D view; checkCIF report

## Figures and Tables

**Table 1:** Hydrogen-bond geometry (Å, °) *Cg*1, *Cg*2, *Cg*3 and *Cg*4 are the centroids of the C11–C15/C20, C21–C25/C30, Pd1/Cl1/Pd1′/Cl1′ and C1/C2/C7–C10 rings, respectively.

*D*—H⋯*A*	*D*—H	H⋯*A*	*D*⋯*A*	*D*—H⋯*A*
C10—H10⋯Cl3	0.95	2.72	3.536 (7)	145
C31—H31*A*⋯Cl3^i^	0.99	2.52	3.366 (16)	143
C9—H9⋯*Cg*1^ii^	0.95	2.83	3.666 (5)	148
C18—H18⋯*Cg*2^iii^	0.95	2.91	3.788 (6)	154
C26—H26⋯*Cg*3^iv^	0.95	2.59	3.535 (5)	172
C31—H31*B*⋯*Cg*4^ii^	0.99	2.75	3.632 (15)	148

Symmetry codes: (i) 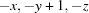; (ii) 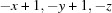; (iii) 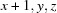; (iv) 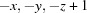.

**Fig. 1.**
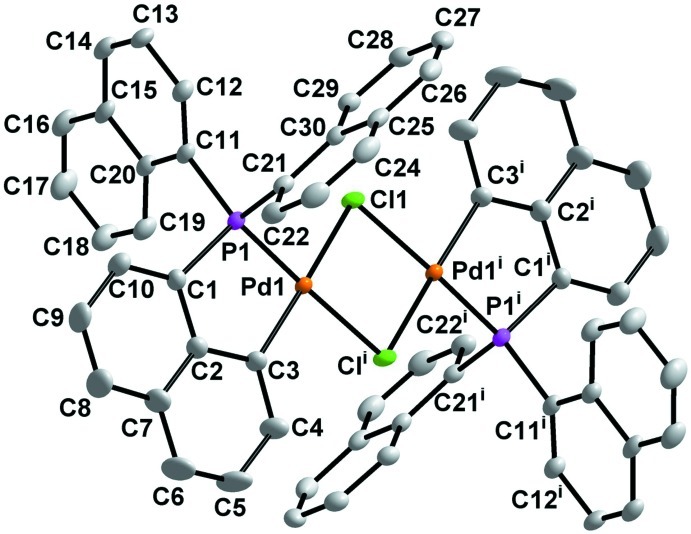
A view of the title complex, showing the atom-numbering scheme and 50% probability displacement ellipsoids (H atoms omitted for clarity). Accented lettering indicate atoms generated by symmetry code i = 1-x, -y, 1-z.

**Fig. 2.**
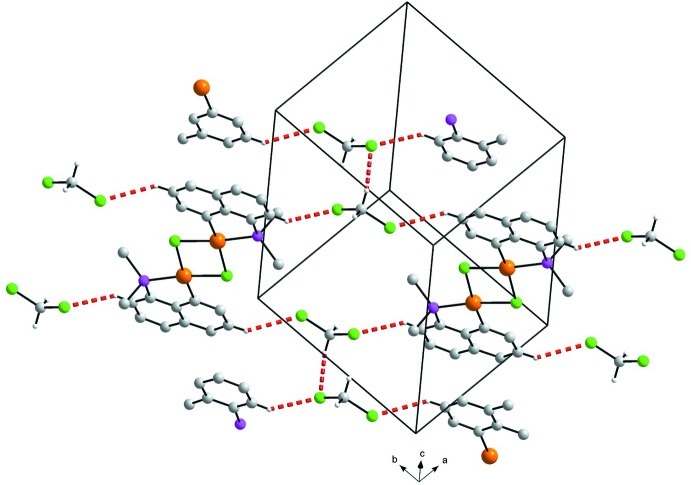
Packing diagram showing the 2-dimentional network generated by C—H···Cl interactions (indicated by red dashed lines) between the metal complex and the dichloromethane solvates (H atoms not involved in interactions are omitted for clarity).

**Fig. 3.**
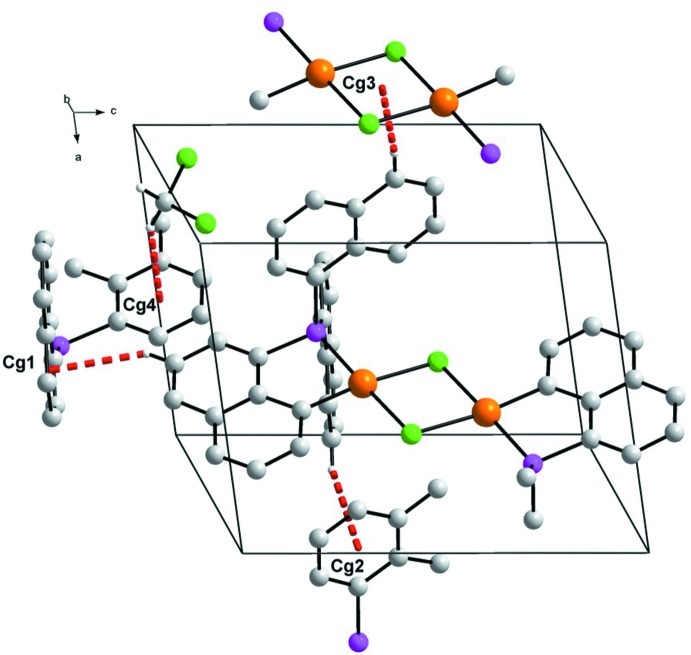
Packing diagram showing the C—H···π interactions (indicated by red dashed lines). H atoms not involved in interactions are omitted for clarity.

**Fig. 4.**
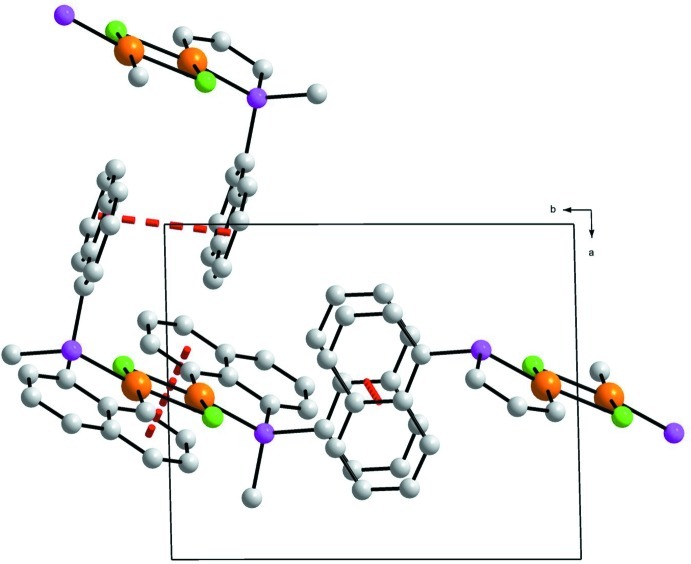
Packing diagram showing the π–π interactions (indicated by red dashed lines). H atoms are omitted for clarity.

## supplementary crystallographic information

### Comment

In the past few decades, phosphapalladacycles have attracted extensive attention due to their activity as catalysts in C—C bond formation scenarios (Dunina *et al.*, 2008, 2009; Bedford *et al.*, 2004; Morales-Morales *et al.*, 2002). [PdCl_2_(*L*)_2_] (*L* = tertiary phosphine, arsine or stibine) complexes can conveniently be prepared by the substitution of 1,5-cyclooctadiene (COD) from [PdCl_2_(COD)]. Reported here is the product of the reaction between tris(naphthalen-1-yl)phosphane and [PdCl_2_(COD)], which shows dimerization of the Pd^II^ metal as well as chelation of one naphthalen-1-yl substituent of each of the phosphane ligands to the Pd dimer.

The title compound (Fig.1) crystallizes in the triclinic space group *P*1 (*Z* = 1), situated around an inversion centre and accompanied by two dichloromethane solvate molecules in the unit cell. The coordination centre for each Pd^II^ centre is distorted due to the strained five membered chelation of the naphthalen-1-yl ligand to each metal centre in a *trans* orientation. This distortion is noted most prominently in the displacement of the P and C donor atoms from the plane formed by the Pd and bridged Cl atoms (C3 and P1 displaced 0.2811 (4) and -0.2508 (12) Å, respectively).

Crystal packing reveals a 2-dimentional network generated by C—H···Cl interactions between the *cyclo*-metalated Pd complex and the dichloromethane solvates (see Fig. 2, table 1). In addition to the above several C—H···π interactions (see Fig. 3, table 1) and π–π stacking (see Fig. 4; centroid to centroid distances = 3.713 (3), 3.850 (4), 3.926 (3) Å) are observed, linking the 2-dimentional layers into 3-dimentional network.

### Experimental

Dichloro(1,5-cyclooctadiene)palladium(II), [PdCl_2_(COD)], was prepared according to the literature procedure of Drew & Doyle (1990). Tris(naphthalen-1-yl)phosphane (15 mg, 0.036 mmol) was dissolved in CH_2_Cl_2_ (5 cm^3^). A solution of [Pd(COD)Cl_2_] (5.2 mg, 0.018 mmol) in CH_2_Cl_2_ (5 cm^3^) was added to the phosphane solution. The mixture was stirred for 6hr at room temperature, after which the solution was left to slowly evaporate giving a yellow powder in 65% yield. Yellow crystals of the title compound suitable for a single-crystal X-ray study were obtained after recrystallization from a CH_2_Cl_2_/DMSO solution.

^31^P NMR (CDCl_3_, 162.0 MHz): δ (p.p.m.) 24.91 (s, 1P).

FTIR (cm^-1^):2199, 2162, 1712, 1630, 1560, 1507, 1252, 1165, 1043, 929, 794, 769, 720, 668, 622.

### Refinement

The aromatic and methylene H atoms were placed in geometrically idealized positions (C—H = 0.95 and 0.99 Å) and allowed to ride on their parent atoms, with *U*_iso_(H) = 1.2*U*_eq_(C). The deepest residual electron-density hole (-2.36 e.Å^3^) is located at 0.7 Å from C31 and the highest peak (1.59 e.Å^3^) 1.12 Å from Cl3, both associated with the solvate molecule and representing no physical meaning.

### Crystal data

**Table:**

[Pd_2_(C_30_H_20_P)_2_Cl_2_]·2CH_2_Cl_2_	*Z* = 1
*M**_r_* = 1276.41	*F*(000) = 640
Triclinic, *P*1	*D*_x_ = 1.651 Mg m^−^^3^
Hall symbol: -P 1	Mo *K*α radiation, λ = 0.71073 Å
*a* = 9.4823 (8) Å	Cell parameters from 9967 reflections
*b* = 11.4272 (9) Å	θ = 2.3–28.4°
*c* = 12.343 (1) Å	µ = 1.12 mm^−^^1^
α = 80.652 (2)°	*T* = 100 K
β = 76.592 (2)°	Needle, yellow
γ = 89.013 (2)°	0.33 × 0.13 × 0.13 mm
*V* = 1283.42 (18) Å^3^	

### Data collection

**Table:**

Bruker APEX DUO 4K-CCD diffractometer	6340 independent reflections
Radiation source: sealed tube	5675 reflections with *I* > 2σ(*I*)
Graphite monochromator	*R*_int_ = 0.030
Detector resolution: 8.4 pixels mm^-1^	θ_max_ = 28.4°, θ_min_ = 1.7°
φ and ω scans	*h* = −12→12
Absorption correction: multi-scan (*SADABS*; Bruker, 2008)	*k* = −15→15
*T*_min_ = 0.709, *T*_max_ = 0.868	*l* = −16→16
40200 measured reflections	

### Refinement

**Table:**

Refinement on *F*^2^	Primary atom site location: structure-invariant direct methods
Least-squares matrix: full	Secondary atom site location: difference Fourier map
*R*[*F*^2^ > 2σ(*F*^2^)] = 0.052	Hydrogen site location: inferred from neighbouring sites
*wR*(*F*^2^) = 0.154	H-atom parameters constrained
*S* = 1.07	*w* = 1/[σ^2^(*F*_o_^2^) + (0.0813*P*)^2^ + 7.0269*P*] where *P* = (*F*_o_^2^ + 2*F*_c_^2^)/3
6340 reflections	(Δ/σ)_max_ < 0.001
325 parameters	Δρ_max_ = 1.59 e Å^−^^3^
0 restraints	Δρ_min_ = −2.36 e Å^−^^3^

### Special details

**Table:**

Experimental. The intensity data was collected on a Bruker Apex DUO 4 K CCD diffractometer using an exposure time of 10 s/frame. A total of 3976 frames were collected with a frame width of 0.5° covering up to θ = 28.38° with 98.6% completeness accomplished.
Geometry. All e.s.d.'s (except the e.s.d. in the dihedral angle between two l.s. planes) are estimated using the full covariance matrix. The cell e.s.d.'s are taken into account individually in the estimation of e.s.d.'s in distances, angles and torsion angles; correlations between e.s.d.'s in cell parameters are only used when they are defined by crystal symmetry. An approximate (isotropic) treatment of cell e.s.d.'s is used for estimating e.s.d.'s involving l.s. planes.
Refinement. Refinement of *F*^2^ against ALL reflections. The weighted *R*-factor *wR* and goodness of fit *S* are based on *F*^2^, conventional *R*-factors *R* are based on *F*, with *F* set to zero for negative *F*^2^. The threshold expression of *F*^2^ > σ(*F*^2^) is used only for calculating *R*-factors(gt) *etc*. and is not relevant to the choice of reflections for refinement. *R*-factors based on *F*^2^ are statistically about twice as large as those based on *F*, and *R*- factors based on ALL data will be even larger.

### Fractional atomic coordinates and isotropic or equivalent isotropic displacement parameters (Å^2^)

**Table:**

	*x*	*y*	*z*	*U*_iso_*/*U*_eq_	
Pd1	0.48238 (3)	0.07664 (2)	0.36232 (2)	0.01412 (11)	
P1	0.37614 (12)	0.23315 (9)	0.28438 (8)	0.0156 (2)	
Cl1	0.42468 (11)	0.10775 (9)	0.55808 (8)	0.0192 (2)	
Cl2	0.0088 (5)	0.7412 (4)	0.0879 (3)	0.1348 (16)	
Cl3	0.1322 (4)	0.5129 (4)	0.0838 (4)	0.1423 (16)	
C3	0.5601 (5)	0.0555 (4)	0.2007 (4)	0.0222 (9)	
C2	0.5448 (6)	0.1477 (4)	0.1105 (4)	0.0252 (9)	
C1	0.4615 (5)	0.2484 (4)	0.1354 (4)	0.0230 (9)	
C21	0.1836 (5)	0.1992 (4)	0.2977 (4)	0.0181 (8)	
C30	0.0811 (5)	0.1809 (3)	0.4047 (3)	0.0179 (8)	
C25	−0.0606 (5)	0.1358 (4)	0.4107 (4)	0.0212 (8)	
C24	−0.0963 (6)	0.1117 (4)	0.3107 (4)	0.0278 (10)	
H24	−0.1899	0.0806	0.3144	0.033*	
C23	0.0024 (6)	0.1326 (4)	0.2095 (4)	0.0272 (10)	
H23	−0.0237	0.1178	0.143	0.033*	
C22	0.1425 (5)	0.1759 (4)	0.2030 (4)	0.0234 (9)	
H22	0.2103	0.1893	0.132	0.028*	
C26	−0.1636 (5)	0.1163 (4)	0.5160 (4)	0.0271 (10)	
H26	−0.2582	0.0874	0.5194	0.033*	
C27	−0.1295 (5)	0.1381 (4)	0.6122 (4)	0.0272 (10)	
H27	−0.1991	0.1233	0.6822	0.033*	
C28	0.0099 (5)	0.1829 (4)	0.6070 (4)	0.0243 (9)	
H28	0.0336	0.1983	0.6742	0.029*	
C29	0.1120 (5)	0.2047 (4)	0.5068 (4)	0.0197 (8)	
H29	0.2046	0.2361	0.5054	0.024*	
C11	0.3889 (5)	0.3794 (3)	0.3228 (3)	0.0183 (8)	
C20	0.5266 (5)	0.4266 (4)	0.3270 (3)	0.0199 (8)	
C19	0.6584 (5)	0.3643 (4)	0.3056 (4)	0.0249 (9)	
H19	0.6575	0.2862	0.2885	0.03*	
C18	0.7875 (6)	0.4152 (4)	0.3092 (5)	0.0324 (11)	
H18	0.8741	0.3715	0.2954	0.039*	
C17	0.7928 (6)	0.5312 (5)	0.3332 (5)	0.0340 (11)	
H17	0.8826	0.5658	0.3345	0.041*	
C16	0.6669 (6)	0.5941 (4)	0.3549 (4)	0.0277 (10)	
H16	0.6705	0.6722	0.3714	0.033*	
C15	0.5323 (6)	0.5443 (4)	0.3528 (4)	0.0227 (9)	
C14	0.4021 (6)	0.6095 (4)	0.3743 (4)	0.0252 (9)	
H14	0.4053	0.6867	0.393	0.03*	
C13	0.2732 (6)	0.5630 (4)	0.3684 (4)	0.0260 (9)	
H13	0.1878	0.6083	0.3822	0.031*	
C12	0.2659 (5)	0.4472 (4)	0.3418 (4)	0.0227 (9)	
H12	0.1757	0.416	0.337	0.027*	
C10	0.4477 (6)	0.3402 (4)	0.0525 (4)	0.0314 (11)	
H10	0.393	0.4075	0.0712	0.038*	
C9	0.5155 (8)	0.3341 (5)	−0.0616 (4)	0.0399 (14)	
H9	0.5063	0.3975	−0.1196	0.048*	
C8	0.5943 (9)	0.2369 (5)	−0.0882 (4)	0.0468 (16)	
H8	0.6375	0.2332	−0.1651	0.056*	
C7	0.6129 (7)	0.1420 (5)	−0.0042 (4)	0.0373 (13)	
C6	0.6978 (9)	0.0412 (6)	−0.0281 (4)	0.0526 (19)	
H6	0.7444	0.0351	−0.1039	0.063*	
C5	0.7122 (9)	−0.0463 (6)	0.0572 (5)	0.0504 (18)	
H5	0.7701	−0.1125	0.0402	0.061*	
C4	0.6423 (6)	−0.0409 (5)	0.1719 (4)	0.0323 (11)	
H4	0.6522	−0.1045	0.2293	0.039*	
C31	0.1100 (14)	0.6358 (15)	0.0065 (11)	0.116 (5)	
H31A	0.0579	0.6198	−0.0506	0.139*	
H31B	0.2061	0.6711	−0.0341	0.139*	

### Atomic displacement parameters (Å^2^)

**Table:**

	*U*^11^	*U*^22^	*U*^33^	*U*^12^	*U*^13^	*U*^23^
Pd1	0.01857 (17)	0.01053 (16)	0.01332 (16)	0.00039 (11)	−0.00303 (11)	−0.00303 (10)
P1	0.0217 (5)	0.0111 (4)	0.0135 (4)	0.0009 (4)	−0.0031 (4)	−0.0021 (3)
Cl1	0.0273 (5)	0.0162 (4)	0.0152 (4)	0.0083 (4)	−0.0056 (4)	−0.0051 (3)
Cl2	0.137 (3)	0.177 (4)	0.0702 (17)	0.055 (3)	−0.0036 (18)	0.004 (2)
Cl3	0.098 (2)	0.131 (3)	0.182 (4)	0.005 (2)	−0.037 (3)	0.026 (3)
C3	0.033 (2)	0.0179 (19)	0.0147 (18)	0.0011 (17)	−0.0013 (16)	−0.0054 (15)
C2	0.037 (3)	0.020 (2)	0.0160 (19)	−0.0006 (18)	0.0004 (17)	−0.0045 (16)
C1	0.033 (2)	0.0167 (19)	0.0162 (18)	−0.0011 (17)	0.0009 (16)	−0.0023 (15)
C21	0.021 (2)	0.0132 (17)	0.0208 (19)	0.0030 (15)	−0.0060 (15)	−0.0036 (14)
C30	0.022 (2)	0.0120 (17)	0.0199 (18)	0.0023 (15)	−0.0063 (15)	−0.0021 (14)
C25	0.021 (2)	0.0129 (18)	0.030 (2)	0.0025 (15)	−0.0073 (17)	−0.0042 (16)
C24	0.029 (2)	0.020 (2)	0.040 (3)	0.0016 (18)	−0.017 (2)	−0.0067 (18)
C23	0.037 (3)	0.023 (2)	0.029 (2)	0.0030 (19)	−0.019 (2)	−0.0101 (18)
C22	0.033 (2)	0.020 (2)	0.0189 (19)	0.0044 (18)	−0.0092 (17)	−0.0065 (16)
C26	0.021 (2)	0.018 (2)	0.039 (3)	0.0015 (16)	−0.0007 (18)	−0.0025 (18)
C27	0.026 (2)	0.020 (2)	0.029 (2)	0.0052 (17)	0.0047 (18)	−0.0029 (17)
C28	0.030 (2)	0.020 (2)	0.022 (2)	0.0069 (17)	−0.0052 (17)	−0.0054 (16)
C29	0.022 (2)	0.0166 (18)	0.0212 (19)	0.0023 (15)	−0.0053 (16)	−0.0060 (15)
C11	0.029 (2)	0.0115 (17)	0.0139 (17)	0.0004 (15)	−0.0051 (15)	−0.0019 (13)
C20	0.031 (2)	0.0129 (18)	0.0161 (18)	0.0007 (16)	−0.0056 (16)	−0.0024 (14)
C19	0.031 (2)	0.0147 (19)	0.030 (2)	0.0002 (17)	−0.0086 (18)	−0.0056 (16)
C18	0.033 (3)	0.022 (2)	0.043 (3)	0.001 (2)	−0.010 (2)	−0.006 (2)
C17	0.038 (3)	0.023 (2)	0.045 (3)	−0.005 (2)	−0.016 (2)	−0.006 (2)
C16	0.043 (3)	0.0158 (19)	0.028 (2)	−0.0017 (19)	−0.014 (2)	−0.0059 (17)
C15	0.040 (3)	0.0112 (17)	0.0172 (18)	0.0013 (17)	−0.0083 (17)	−0.0014 (14)
C14	0.042 (3)	0.0126 (18)	0.021 (2)	0.0024 (18)	−0.0071 (18)	−0.0038 (15)
C13	0.037 (3)	0.0133 (19)	0.027 (2)	0.0066 (17)	−0.0046 (19)	−0.0042 (16)
C12	0.033 (2)	0.0147 (19)	0.0203 (19)	0.0017 (17)	−0.0056 (17)	−0.0025 (15)
C10	0.051 (3)	0.018 (2)	0.021 (2)	0.005 (2)	−0.002 (2)	−0.0014 (17)
C9	0.071 (4)	0.025 (2)	0.017 (2)	0.000 (3)	−0.002 (2)	0.0041 (18)
C8	0.083 (5)	0.030 (3)	0.018 (2)	0.007 (3)	0.003 (3)	0.001 (2)
C7	0.060 (4)	0.030 (3)	0.015 (2)	0.007 (2)	0.003 (2)	−0.0045 (18)
C6	0.088 (5)	0.045 (3)	0.015 (2)	0.025 (3)	0.006 (3)	−0.007 (2)
C5	0.084 (5)	0.040 (3)	0.023 (2)	0.027 (3)	−0.001 (3)	−0.014 (2)
C4	0.049 (3)	0.030 (2)	0.017 (2)	0.013 (2)	−0.005 (2)	−0.0074 (18)
C31	0.095 (9)	0.176 (14)	0.092 (8)	0.047 (9)	−0.043 (7)	−0.044 (9)

### Geometric parameters (Å, º)

**Table:**

Pd1—C3	2.013 (4)	C11—C12	1.385 (6)
Pd1—P1	2.2288 (10)	C11—C20	1.436 (6)
Pd1—Cl1^i^	2.4180 (10)	C20—C19	1.420 (6)
Pd1—Cl1	2.4337 (10)	C20—C15	1.435 (6)
P1—C1	1.811 (4)	C19—C18	1.377 (7)
P1—C11	1.823 (4)	C19—H19	0.95
P1—C21	1.838 (4)	C18—C17	1.408 (7)
Cl1—Pd1^i^	2.4180 (10)	C18—H18	0.95
Cl2—C31	1.813 (14)	C17—C16	1.378 (8)
Cl3—C31	1.604 (15)	C17—H17	0.95
C3—C4	1.384 (6)	C16—C15	1.414 (7)
C3—C2	1.435 (6)	C16—H16	0.95
C2—C1	1.419 (6)	C15—C14	1.425 (7)
C2—C7	1.424 (6)	C14—C13	1.363 (7)
C1—C10	1.368 (6)	C14—H14	0.95
C21—C22	1.380 (6)	C13—C12	1.420 (6)
C21—C30	1.433 (6)	C13—H13	0.95
C30—C29	1.426 (6)	C12—H12	0.95
C30—C25	1.429 (6)	C10—C9	1.418 (7)
C25—C26	1.421 (6)	C10—H10	0.95
C25—C24	1.422 (7)	C9—C8	1.370 (8)
C24—C23	1.365 (7)	C9—H9	0.95
C24—H24	0.95	C8—C7	1.413 (8)
C23—C22	1.406 (7)	C8—H8	0.95
C23—H23	0.95	C7—C6	1.425 (8)
C22—H22	0.95	C6—C5	1.359 (8)
C26—C27	1.362 (7)	C6—H6	0.95
C26—H26	0.95	C5—C4	1.428 (7)
C27—C28	1.409 (7)	C5—H5	0.95
C27—H27	0.95	C4—H4	0.95
C28—C29	1.372 (6)	C31—H31A	0.99
C28—H28	0.95	C31—H31B	0.99
C29—H29	0.95		
			
C3—Pd1—P1	83.19 (13)	C19—C20—C15	117.8 (4)
C3—Pd1—Cl1^i^	95.05 (13)	C19—C20—C11	123.8 (4)
P1—Pd1—Cl1^i^	173.01 (4)	C15—C20—C11	118.4 (4)
C3—Pd1—Cl1	171.70 (15)	C18—C19—C20	121.1 (4)
P1—Pd1—Cl1	100.16 (3)	C18—C19—H19	119.5
Cl1^i^—Pd1—Cl1	82.51 (3)	C20—C19—H19	119.5
C1—P1—C11	105.94 (19)	C19—C18—C17	120.9 (5)
C1—P1—C21	106.2 (2)	C19—C18—H18	119.5
C11—P1—C21	107.83 (19)	C17—C18—H18	119.5
C1—P1—Pd1	103.99 (15)	C16—C17—C18	119.6 (5)
C11—P1—Pd1	121.53 (14)	C16—C17—H17	120.2
C21—P1—Pd1	110.23 (13)	C18—C17—H17	120.2
Pd1^i^—Cl1—Pd1	97.49 (3)	C17—C16—C15	121.0 (4)
C4—C3—C2	117.3 (4)	C17—C16—H16	119.5
C4—C3—Pd1	122.1 (3)	C15—C16—H16	119.5
C2—C3—Pd1	120.2 (3)	C16—C15—C14	121.1 (4)
C1—C2—C7	118.6 (4)	C16—C15—C20	119.6 (4)
C1—C2—C3	119.6 (4)	C14—C15—C20	119.3 (5)
C7—C2—C3	121.7 (4)	C13—C14—C15	121.0 (4)
C10—C1—C2	121.6 (4)	C13—C14—H14	119.5
C10—C1—P1	127.1 (4)	C15—C14—H14	119.5
C2—C1—P1	111.2 (3)	C14—C13—C12	120.4 (4)
C22—C21—C30	119.6 (4)	C14—C13—H13	119.8
C22—C21—P1	117.7 (3)	C12—C13—H13	119.8
C30—C21—P1	122.1 (3)	C11—C12—C13	120.6 (5)
C29—C30—C25	117.5 (4)	C11—C12—H12	119.7
C29—C30—C21	123.9 (4)	C13—C12—H12	119.7
C25—C30—C21	118.7 (4)	C1—C10—C9	119.6 (5)
C26—C25—C24	121.0 (4)	C1—C10—H10	120.2
C26—C25—C30	119.7 (4)	C9—C10—H10	120.2
C24—C25—C30	119.3 (4)	C8—C9—C10	120.0 (5)
C23—C24—C25	120.7 (5)	C8—C9—H9	120
C23—C24—H24	119.6	C10—C9—H9	120
C25—C24—H24	119.6	C9—C8—C7	121.7 (5)
C24—C23—C22	120.3 (4)	C9—C8—H8	119.2
C24—C23—H23	119.8	C7—C8—H8	119.2
C22—C23—H23	119.8	C8—C7—C2	118.5 (5)
C21—C22—C23	121.3 (4)	C8—C7—C6	123.3 (5)
C21—C22—H22	119.3	C2—C7—C6	118.2 (5)
C23—C22—H22	119.3	C5—C6—C7	120.1 (5)
C27—C26—C25	121.3 (5)	C5—C6—H6	119.9
C27—C26—H26	119.4	C7—C6—H6	119.9
C25—C26—H26	119.4	C6—C5—C4	121.5 (5)
C26—C27—C28	119.5 (4)	C6—C5—H5	119.3
C26—C27—H27	120.3	C4—C5—H5	119.3
C28—C27—H27	120.3	C3—C4—C5	121.1 (5)
C29—C28—C27	121.2 (4)	C3—C4—H4	119.4
C29—C28—H28	119.4	C5—C4—H4	119.4
C27—C28—H28	119.4	Cl3—C31—Cl2	112.5 (8)
C28—C29—C30	121.0 (4)	Cl3—C31—H31A	109.1
C28—C29—H29	119.5	Cl2—C31—H31A	109.1
C30—C29—H29	119.5	Cl3—C31—H31B	109.1
C12—C11—C20	120.2 (4)	Cl2—C31—H31B	109.1
C12—C11—P1	119.3 (3)	H31A—C31—H31B	107.8
C20—C11—P1	120.4 (3)		
			
C3—Pd1—P1—C1	−11.1 (2)	C25—C26—C27—C28	1.1 (7)
Cl1—Pd1—P1—C1	161.18 (17)	C26—C27—C28—C29	−0.1 (7)
C3—Pd1—P1—C11	−130.1 (2)	C27—C28—C29—C30	−1.0 (7)
Cl1—Pd1—P1—C11	42.19 (17)	C25—C30—C29—C28	1.1 (6)
C3—Pd1—P1—C21	102.4 (2)	C21—C30—C29—C28	−178.7 (4)
Cl1—Pd1—P1—C21	−85.31 (15)	C1—P1—C11—C12	106.0 (4)
P1—Pd1—Cl1—Pd1^i^	173.47 (4)	C21—P1—C11—C12	−7.4 (4)
Cl1^i^—Pd1—Cl1—Pd1^i^	0	Pd1—P1—C11—C12	−135.9 (3)
P1—Pd1—C3—C4	−175.6 (5)	C1—P1—C11—C20	−70.5 (4)
Cl1^i^—Pd1—C3—C4	−2.4 (5)	C21—P1—C11—C20	176.1 (3)
P1—Pd1—C3—C2	11.2 (4)	Pd1—P1—C11—C20	47.6 (4)
Cl1^i^—Pd1—C3—C2	−175.6 (4)	C12—C11—C20—C19	−178.3 (4)
C4—C3—C2—C1	179.9 (5)	P1—C11—C20—C19	−1.8 (6)
Pd1—C3—C2—C1	−6.5 (7)	C12—C11—C20—C15	0.8 (6)
C4—C3—C2—C7	−1.0 (8)	P1—C11—C20—C15	177.3 (3)
Pd1—C3—C2—C7	172.6 (4)	C15—C20—C19—C18	−0.1 (7)
C7—C2—C1—C10	−1.2 (8)	C11—C20—C19—C18	178.9 (4)
C3—C2—C1—C10	177.9 (5)	C20—C19—C18—C17	−0.6 (8)
C7—C2—C1—P1	176.5 (4)	C19—C18—C17—C16	0.9 (8)
C3—C2—C1—P1	−4.4 (6)	C18—C17—C16—C15	−0.3 (8)
C11—P1—C1—C10	−42.0 (6)	C17—C16—C15—C14	−179.5 (5)
C21—P1—C1—C10	72.5 (5)	C17—C16—C15—C20	−0.4 (7)
Pd1—P1—C1—C10	−171.1 (5)	C19—C20—C15—C16	0.6 (6)
C11—P1—C1—C2	140.5 (4)	C11—C20—C15—C16	−178.5 (4)
C21—P1—C1—C2	−105.0 (4)	C19—C20—C15—C14	179.8 (4)
Pd1—P1—C1—C2	11.4 (4)	C11—C20—C15—C14	0.6 (6)
C1—P1—C21—C22	8.5 (4)	C16—C15—C14—C13	177.7 (4)
C11—P1—C21—C22	121.7 (3)	C20—C15—C14—C13	−1.4 (7)
Pd1—P1—C21—C22	−103.6 (3)	C15—C14—C13—C12	0.7 (7)
C1—P1—C21—C30	179.9 (3)	C20—C11—C12—C13	−1.5 (6)
C11—P1—C21—C30	−66.9 (4)	P1—C11—C12—C13	−178.0 (3)
Pd1—P1—C21—C30	67.9 (3)	C14—C13—C12—C11	0.8 (7)
C22—C21—C30—C29	−178.7 (4)	C2—C1—C10—C9	1.3 (9)
P1—C21—C30—C29	10.1 (6)	P1—C1—C10—C9	−176.0 (5)
C22—C21—C30—C25	1.5 (6)	C1—C10—C9—C8	0.0 (10)
P1—C21—C30—C25	−169.7 (3)	C10—C9—C8—C7	−1.3 (11)
C29—C30—C25—C26	−0.2 (6)	C9—C8—C7—C2	1.3 (11)
C21—C30—C25—C26	179.6 (4)	C9—C8—C7—C6	−178.0 (7)
C29—C30—C25—C24	179.5 (4)	C1—C2—C7—C8	−0.1 (9)
C21—C30—C25—C24	−0.6 (6)	C3—C2—C7—C8	−179.2 (6)
C26—C25—C24—C23	178.9 (4)	C1—C2—C7—C6	179.2 (6)
C30—C25—C24—C23	−0.9 (7)	C3—C2—C7—C6	0.1 (9)
C25—C24—C23—C22	1.5 (7)	C8—C7—C6—C5	179.3 (8)
C30—C21—C22—C23	−1.0 (6)	C2—C7—C6—C5	0.0 (12)
P1—C21—C22—C23	170.7 (3)	C7—C6—C5—C4	0.8 (13)
C24—C23—C22—C21	−0.6 (7)	C2—C3—C4—C5	1.8 (9)
C24—C25—C26—C27	179.4 (4)	Pd1—C3—C4—C5	−171.6 (5)
C30—C25—C26—C27	−0.9 (6)	C6—C5—C4—C3	−1.8 (11)

Symmetry code: (i) −*x*+1, −*y*, −*z*+1.

### Hydrogen-bond geometry (Å, º)

*Cg*1, *Cg*2, *Cg*3 and *Cg*4 are the centroids of the C11–C15/C20, C21–C25/C30, Pd1/Cl1/Pd1'/Cl1' and C1/C2/C7–C10 rings, respectively.

**Table:**

*D*—H···*A*	*D*—H	H···*A*	*D*···*A*	*D*—H···*A*
C10—H10···Cl3	0.95	2.72	3.536 (7)	145
C31—H31*A*···Cl3^ii^	0.99	2.52	3.366 (16)	143
C9—H9···*Cg*1^iii^	0.95	2.83	3.666 (5)	148
C18—H18···*Cg*2^iv^	0.95	2.91	3.788 (6)	154
C26—H26···*Cg*3^v^	0.95	2.59	3.535 (5)	172
C31—H31*B*···*Cg*4^iii^	0.99	2.75	3.632 (15)	148

Symmetry codes: (ii) −*x*, −*y*+1, −*z*; (iii) −*x*+1, −*y*+1, −*z*; (iv) *x*+1, *y*, *z*; (v) −*x*, −*y*, −*z*+1.
